# Positive predictive value of non-invasive prenatal screening for fetal chromosome disorders using cell-free DNA in maternal serum: independent clinical experience of a tertiary referral center

**DOI:** 10.1186/s12916-015-0374-8

**Published:** 2015-06-02

**Authors:** Whitney A. Neufeld-Kaiser, Edith Y. Cheng, Yajuan J. Liu

**Affiliations:** Department of Pathology, Cytogenetics and Genomics Laboratory, University of Washington School of Medicine, Box 357470, HSB H474B, 1959 NE Pacific Street, Seattle, WA 98195-7470 USA; Maternal Fetal Medicine and Medical Genetics, Departments of Obstetrics and Gyencology and Internal Medicine, University of Washington School of Medicine, Seattle, WA USA

**Keywords:** Cell-free DNA, NIPS, NIPT, Aneuploidy, Positive predictive value, Diagnostic genetic test

## Abstract

**Background:**

Non-invasive prenatal screening (NIPS) for fetal chromosome abnormalities using cell-free deoxyribonucleic acid (cfDNA) in maternal serum has significantly influenced prenatal diagnosis of fetal aneuploidies since becoming clinically available in the fall of 2011. High sensitivity and specificity have been reported in multiple publications, nearly all of which have been sponsored by the commercial performing laboratories. Once results are returned, positive and negative predictive values (PPVs, NPVs) are the performance metrics most relevant to clinical management. The purpose of this report is to present independent data on the PPVs of NIPS in actual clinical practice.

**Methods:**

Charts were retrospectively reviewed for patients who had NIPS and were seen March 2012 to December 2013 in a tertiary academic referral center. NIPS results were compared to diagnostic genetic test results, fetal ultrasound results, and clinical phenotype/outcomes. The PPV was calculated using standard epidemiological methods. Correlation between screen results and both maternal age at delivery and gestational age at time of screening was assessed using Wilcoxon’s rank sum test.

**Results:**

Of 632 patients undergoing NIPS, 92 % of tests were performed in one of the four major commercial laboratories offering testing. However, all four laboratories are represented in both the normal and abnormal results groups. There were 55 abnormal NIPS results. Forty-one of 55 abnormal NIPS results were concordant with abnormal fetal outcomes, 12 were discordant, and 2 were undetermined. The PPV for all conditions included in the screen was 77.4 % (95 % CI, 63.4 – 87.3). Of 578 patients with normal NIPS results, normal pregnancy outcome was confirmed for 156 (27 %) patients. This incomplete follow-up of normal NIPS results does not affect PPV calculations, but it did preclude calculations of sensitivity, specificity, and NPV. Maternal age at delivery was significantly lower for patients with abnormal discordant results, compared to patients with abnormal concordant results (*P* = 0.034). Gestational age at time of screening was not associated with concordance of screen results (*P* = 0.722).

**Conclusions:**

The experience of using NIPS in clinical practice confirms that abnormal results cannot be considered diagnostic. Pre-test counseling should emphasize this. Diagnostic genetic testing should always be offered following abnormal NIPS results.

## Background

Cell-free deoxyribonucleic acid (cfDNA) refers to fragments of DNA circulating freely in the plasma. The fragments are most frequently 150-180 base pairs in length [1, 2] and derive mostly from apoptotic cells [2]. During pregnancy, placental cytotrophoblastic cells are shed into maternal circulation and contribute to the cfDNA pool in the maternal bloodstream [3]. After 10 weeks gestation, an average of 10 % of maternal cfDNA is placental in origin [4]. Non-invasive prenatal screening (NIPS) for fetal chromosome abnormalities is based on either massively parallel sequencing [5–11] or analysis of single nucleotide polymorphism (SNP) patterns [12, 13] from cfDNA in maternal serum. Very high sensitivity (98.6 - 100 %) and specificity (99.7 - 100 %) have been reported in multiple clinical validation studies of NIPS for Down syndrome (DS) [6, 9, 10, 12, 14]. Somewhat lower sensitivities and specificities are seen when screening for trisomy 18 (T18), trisomy 13 (T13), and the sex chromosome aneuploidies (SCAs: 45,X; 47,XXX; 47,XXY; 47,XYY) [7–11, 13–17].

Although sensitivity and specificity are important performance metrics, positive predictive value (PPV) and negative predictive value (NPV) become more clinically relevant after results have returned. Although some publications sponsored by commercial laboratories performing NIPS have included PPV and NPV [10, 14, 18, 19], there is minimal independent data available on the performance of NIPS in actual clinical practice [20–23].

Here we present our first two years’ experience with NIPS in a tertiary referral center. Performance was evaluated by calculating standard metrics such as PPV. Underlying biological causes for discordant results were determined where possible. Some discordant or unusual cases are described in detail. Benefits and limitations of using NIPS in clinical practice and recommendations for follow-up of abnormal results are discussed.

## Methods

This study was approved by the Human Subjects Division (Application no. 47,683) and the Institutional Review Board (IRB) at the University of Washington Medical Center (UWMC). The requirement to obtain written consent and the requirement for Health Insurance Portability and Accountability Act (HIPAA) authorization were both waived by the IRB. Medical records were retrospectively reviewed for 632 consecutive patients who had NIPS and were seen between March 2012 and December 2013 in the Prenatal Genetics and Fetal Therapy (PGFT) Program, a tertiary referral center for prenatal genetic counseling and prenatal diagnosis located at UWMC. Patient age at delivery, gestational age at screening, indication for screening, and maternal serum screen results were extracted from outpatient clinic notes. Pregnancy outcomes were extracted from delivery summaries. Fetal ultrasound results were extracted from radiology records; diagnostic genetic test results were extracted from cytogenetics records; and NIPS results were extracted from laboratory records. Of the 632 patients undergoing NIPS, 92 % of the tests were performed in one of the four major commercial laboratories offering testing during this timeframe.

Patients with normal NIPS results typically declined prenatal diagnostic genetic testing. If not already completed, maternal serum alpha-fetoprotein (AFP) and fetal anatomy ultrasound were recommended, and the patient was referred back to her primary obstetrical provider for routine care. Patients with abnormal NIPS results were offered high-resolution fetal ultrasound interpreted by either a UWMC perinatologist or a UWMC radiologist specializing in fetal anatomical imaging, genetic counseling, and prenatal diagnostic genetic testing via chorionic villus sampling (CVS) or amniocentesis. When the latter was declined, postnatal testing was recommended. Maternal chromosome analysis was added to our recommendations several months after we started to offer NIPS. For all patients, follow-up care recommendations were communicated verbally to the patient at the time of the NIPS results disclosure and/or genetic counseling consult, and in writing to the referring provider.

Normal NIPS results were defined as concordant when either diagnostic genetic test results matched the screen results or, for patients declining prenatal diagnostic genetic testing and delivering at UWMC, when normal pregnancy outcome was confirmed by review of delivery records. Newborn exams were performed by pediatricians. For patients with normal NIPS results who declined prenatal diagnostic genetic testing and delivered elsewhere, pregnancy outcomes were not confirmed. Abnormal NIPS results were defined as concordant when either diagnostic genetic test results matched the screen results or when multiple other clinical findings (such as fetal ultrasound abnormalities) corroborated the screen results. Nuchal translucency (NT) was defined as abnormal when the DS likelihood ratio was ≥ 2 [24]. Patients who declined diagnostic genetic testing and were lost to follow-up with incomplete clinical information were not included in calculations of test performance. Diagnostic genetic test methods included interphase fluorescence in situ hybridization (IFISH), karyotyping, and cytogenomic microarray analysis (CMA). Diagnostic genetic testing was performed by the University of Washington Cytogenetics and Genomics Laboratory unless otherwise stated.

The R statistical software package (version R 2.12.0) [25] and Excel were used for all statistical analyses. For categorical data, frequencies or percentages with 95 % confidence intervals were derived. For quantitative data, means with standard deviations, medians with minimum and maximum values, and frequencies were calculated. Wilcoxon’s rank sum test was used to evaluate statistical significance between groups, and *P*-values of ≤0.05 were considered statistically significant. The PPV was calculated with standard methods [26]. The reported performance of NIPS is remarkably similar across all test platforms in studies sponsored by the commercial laboratories [6, 9, 10, 12, 14, 19], so all NIPS results for this cohort were lumped, such that all performing commercial laboratories are represented therein.

## Results

### Patient characteristics

**Fig. 1:**
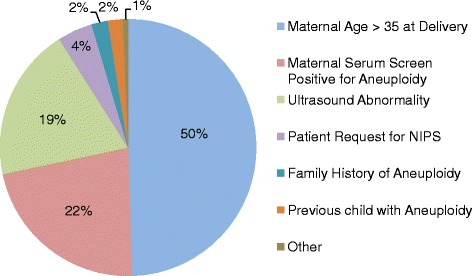
Indications for NIPS. Abbreviations: NIPS, non-invasive prenatal screening

**Fig. 2:**
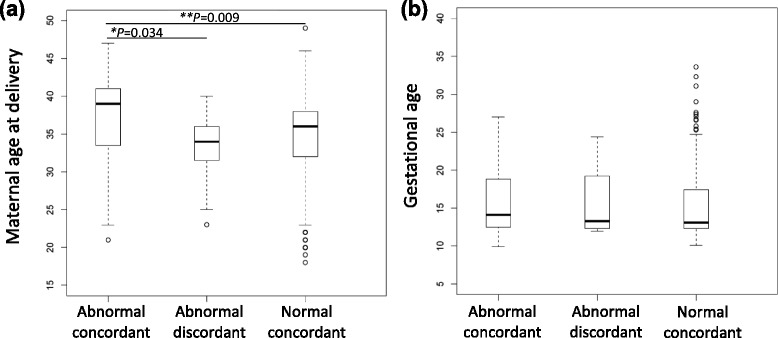
Median and distribution of **a** maternal age at delivery and **b** gestational age at NIPS. *P*-values based on Wilcoxon’s rank sum test (*P < 0.05, **P < 0.01). Abbreviations: NIPS, non-invasive prenatal screening

Of 632 patients, most were at increased risk for fetal aneuploidy as defined by American Congress of Obstetricians and Gynecologists guidelines [27]. Frequent indications for NIPS included maternal age ≥35 at delivery, abnormal serum screen results, fetal ultrasound abnormalities, and family or personal history of a previous child with aneuploidy (Fig. 1). To determine whether concordance of NIPS results was correlated with maternal age at delivery or gestational age at screening, medians and their distributions were plotted and assessed for statistical significance (Fig. 2, Table 1). Maternal age at delivery was significantly older for patients with abnormal concordant results compared to patients with abnormal discordant results (*P* = 0.034) and to patients with normal results (*P* = 0.009). Gestational age at screening ranged from 9.9 to 33.6 weeks and was not significantly associated with concordance of results.

**Table 1:** Maternal age at delivery and gestational age at time of NIPS, stratified by NIPS results

			Median	Mean	SD	*P*-values^a^	
Maternal age (years)	I.	Abnormal concordant	39.00	36.97	6.13	I vs. III	0.009**
	II.	Abnormal discordant	34.00	33.08	4.70	I vs. II	0.034*
	III.	Normal concordant	36.00	34.79	5.16	II vs. III	0.161
Gestational age (weeks)	I.	Abnormal concordant	13.10	14.94	3.74	I vs. III	0.156
	II.	Abnormal discordant	14.10	15.76	4.17	I vs. II	0.722
	III.	Normal concordant	13.25	15.64	3.97	II vs. III	0.627

^a^ *P*-values calculated using Wilcoxon rank sum test

*P < 0.05

**P < 0.01

### PPV of NIPS in an independent clinical setting

**Fig. 3:**
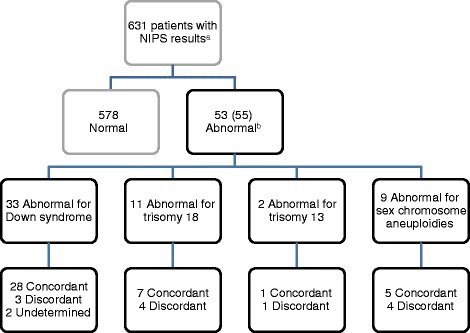
NIPS results for this cohort. ^a^Sex chromosome aneuploidies were included in only 520 patients. ^b^Two patients had abnormal results for two different chromosomes. Abbreviations: NIPS, non-invasive prenatal screening

Figure 3 shows the breakdown of NIPS results for this patient cohort. Of 632 patients offered NIPS, no results were obtained for one patient, giving a test failure rate of 0.16 %. No redraws were needed in this cohort. Fifty-three of 631 (8 %) patients had abnormal NIPS results. This relatively high percentage reflects our high-risk patient population. Two patients had abnormal results for two chromosomes. One had abnormal results for DS and 47,XXX, and one had abnormal results for T18 and DS.

Among 55 abnormal NIPS results, 41 were concordant, 2 were undetermined and lost to follow-up, and 12 were discordant with diagnostic genetic testing and/or clinical phenotype (Fig. 3). Table 2 summarizes the clinical details of the 12 patients with discordant results. The overall PPV for all disorders included in the screen was 77.4 % (95 % CI, 63.4 - 87.3). The highest PPV was for DS (90.3 %, 95 % CI 73.1 - 97.5). NIPS for T18 had the second highest PPV at 63.6 % (95 % CI 31.6 - 87.6). The PPVs for the combined SCAs and for T13 were lowest, at 55.6 % (95 % CI 22.6 - 84.6) and 50 % (95 % CI 2.7 - 97.3), respectively. If the 8 abnormal NIPS results that were compared to phenotype and clinical outcomes, instead of diagnostic genetic test results, are excluded, the overall PPV was 82.9 % (95 % CI 68.6 - 91.9) in this cohort.

**Table 2:** Details of discordant cases

Patient	NIPS result	Other clinical information	Fetal diagnostic genetic test results	Maternal results
31	Abnormal for DS^a^	US at 19.3w multiple anomalies consistent with T18	nuc ish (D21S259x2) on FFPE fetal tissue	Not offered
32	Abnormal for DS	US at 12.3w abnormal NT; US at 20.7w normal; postpartum note “delivered a male infant at 36.6w…birth weight 6 lbs 10 oz….”	Declined	Not offered
33	Abnormal for DS	Paternal first cousin with DS; US at 13.4w normal NT; US at 16.6w normal	46,XY in all 15 amniocyte clones; arr(1-22)*x*2,(XY)x1 on amniocytes	46,XX in all 20 peripheral blood cells analyzed and 30 additional cells screened for 21
41	Abnormal for T18	AMA; FTS pos DS (1 in 43); US at 13.1w normal NT; US at 17.6w and 19.7w normal	Declined	Not offered
42	Abnormal for T18	AMA; US at 19.6w normal	nuc ish(D18Z1x2)[200/200] on uncultured amniocytes; 46,XY in all 52 amniocyte clones	Not offered
43	Abnormal for T18	AMA; US at 12.7w normal NT; US at 20.4w normal	Declined	Not offered
44	Abnormal for T18	US at 19.0w EIF; discharge exam: “Baby does not have any physical exam findings concerning for trisomy 18.”	Declined	Not offered
46	Abnormal for T13	AMA; US at 13.7w normal NT; US at 20.4w normal	nuc ish(RB1x2)[50/50] on uncultured amniocytes; 46,XX in all 15 amniocyte clones	46,XX in all 20 peripheral blood cells analyzed and 50 additional cells screened for 13
51	Abnormal for 45,X	AMA; US at 19.3 normal	nuc ish(DXZ1x2), (DYZ3x0)[191/200]; 46,XX in all 21 amniocyte clones and 100 cells from mass culture	45,X[5]/46,XX[45] in peripheral blood
52	Abnormal for 45,X	AMA; US at 12.3w normal NT; US at 20.3w normal	nuc ish(DXZ1x2)[220/225], (DYZ3x0)[225/225] on uncultured amniocytes; 46,XX in all 16 amniocyte clones analyzed and 22 additional clones screened for 45X	46,XX in all 20 peripheral blood cells analyzed and 30 additional cells screened for X
55	Abnormal for 47,XXX	US at 18.8w unilateral CPC and unilateral renal pelviectasis	46,XX in all 20 cells analyzed from peripheral blood postnatally	46,XX in all 20 peripheral blood cells analyzed and 30 additional cells screened for X
56	Abnormal for 47,XXX	Quad screen pos DS 1 in 15, maternal diabetes and renal failure, US at 21.3w two-vessel cord	46,XX in all 20 cells analyzed from peripheral blood at 7 weeks of age	Not done secondary to chaotic family circumstances

*Abbreviation:* *AMA* advanced maternal age, *CPC* choroid plexus cyst, *DS* Down syndrome, *EIF* echogenic intracardiac focus, *FFPE* formalin-fixed, paraffin-embedded, *FTS* first trimester (maternal serum) screen, *NT* nuchal translucency, *T18* trisomy 18, *T13* trisomy 13, *US* ultrasound

^a^Results were also abnormal for T18

### Sex assessment

NIPS results can also include fetal sex assessment, raising the possibility of normal results that are discordant for sex. We are aware of a single instance of normal NIPS results discordant for fetal sex during this timeframe. Patient 60 was a 26-year-old who presented at 12.4 weeks for routine aneuploidy screening. She had a personal history of congenital unilateral renal agenesis with contralateral damage secondary to reflux. She had undergone a renal transplant from a male donor three years prior. NIPS results were normal and consistent with a male fetus. During pre-test counseling and again when she was informed of the results, she was cautioned that the sex assessment may not be accurate in her case. Fetal anatomy ultrasound at 21.4 weeks showed female genitalia. She was counseled that the likely explanation for the gender discordance was that her cfDNA pool included Y chromosome DNA from the transplanted kidney. She declined amniocentesis for prenatal genetic diagnostic testing. The baby had normal appearing female external genitalia at birth. Karyotyping was not considered indicated and was not done prior to discharge.

### Follow-up after normal NIPS results

**Fig. 4:**
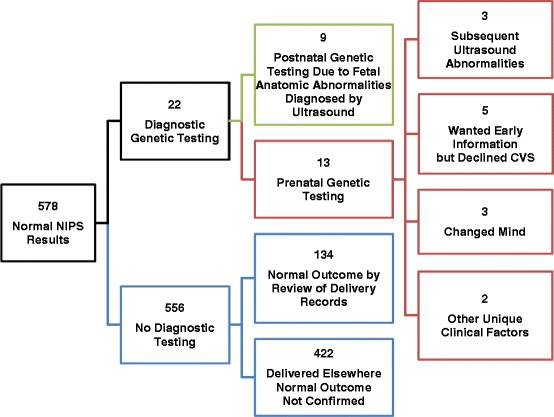
Follow-up after normal NIPS results. Abbreviations: CVS, chorionic villus sampling; NIPS, non-invasive prenatal screening

Of 578 patients with normal NIPS results, 22 (4 %) had diagnostic genetic testing, the results of which were concordant in all cases for the chromosomes screened (Fig. 4). Diagnostic genetic testing was done postnatally on cord blood in 9 of the 22 cases secondary to congenital anomalies detected prenatally by ultrasound. In the other 13 cases, the patient chose to have prenatal testing by amniocentesis. Among these 13 patients, 3 opted for diagnostic testing after subsequent fetal anatomy ultrasound revealed anatomical anomalies; 5 stated an upfront preference for diagnostic genetic testing, but felt CVS was too risky, and used NIPS to assess fetal status at an early gestational age; 3 changed their minds during the prenatal genetic evaluation process and decided they preferred diagnostic testing; and the remaining 2 patients had unusual, unique, clinical circumstances that influenced their decision to pursue prenatal diagnostic genetic testing. Of patients with normal NIPS results who had no diagnostic genetic testing, 134 (23 %) had a normal pregnancy outcome confirmed by review of UWMC delivery records. Pregnancy outcome was not confirmed for the 422 (73 %) patients delivering elsewhere.

### Follow-up after abnormal NIPS results

**Fig. 5:**
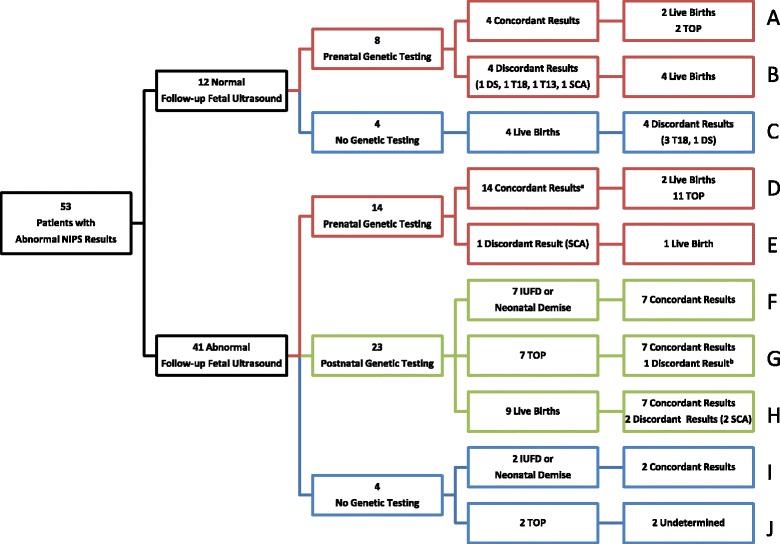
Patient decisions after abnormal NIPS results and clinical outcomes. ^a^One patient had abnormal results for two different chromosomes (DS, XXX); both results were concordant. ^b^One patient had abnormal results for two different chromosomes; one result was concordant (T18) and one was discordant (DS). Abbreviations: DS, Down syndrome; IUFD, intrauterine fetal demise; NIPS, non-invasive prenatal screening; SCA, sex chromosome aneuploidy; T13, trisomy 13; T18, trisomy 18; TOP, termination of pregnancy

Fifty-three of 632 patients had abnormal NIPS results and were offered genetic counseling and follow-up testing. Figure 5 outlines patient decisions and outcomes after abnormal NIPS results. Follow-up fetal ultrasound results were normal in 12 of 53 (23 %) patients with abnormal NIPS results. NIPS results were discordant in 8 of 12 (67 %) patients with normal follow-up fetal ultrasound results (Fig. 5, b and c). Fetal ultrasound anomalies were seen in the other 41 (77 %) patients with abnormal NIPS results. NIPS results were discordant in 4 of 39 (10 %) patients with abnormal follow-up fetal ultrasound results (Fig. 5e, g, and h), excluding the 2 undetermined cases (Fig. 5j).

**Fig. 6:**
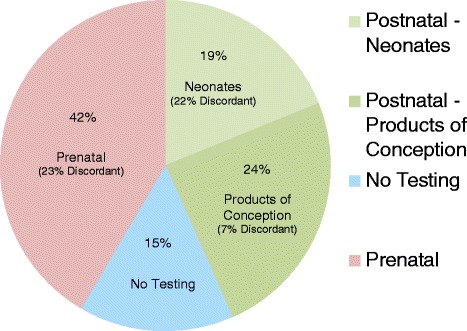
Diagnostic genetic testing after abnormal NIPS results. Abbreviations: NIPS, non-invasive prenatal screening

A genetic diagnosis was obtained in 45 (85 %) patients (Fig. 6). Twenty-two of 45 patients (49 %) chose prenatal diagnostic genetic testing (Fig. 5). Thirteen of these patients had concordant abnormal karyotype results and chose termination of pregnancy (TOP) (Fig. 5, a and d). The other 9, including 5 patients with discordant normal karyotype results (Fig. 5, b and e) and 4 patients with concordant abnormal karyotype results (Fig. 5, a and d), continued to term. Twenty-three of 45 patients (51 %) declined prenatal diagnostic testing, and postnatal testing was done instead (Fig. 5). Seven patients had an intrauterine fetal demise (IUFD) or neonatal demise (Fig. 5f). Seven patients opted for TOP after follow-up fetal anatomy ultrasound revealed anomalies consistent with the condition suggested by their NIPS results. Among these 14 patients, NIPS results were concordant with diagnostic genetic testing of fetal tissue in 14 of 15 instances. The single discordance occurred in a patient whose NIPS result was abnormal for both T18 and DS (Fig. 5g), as IFISH of fetal tissue showed two signals for chromosome 21 and three signals for chromosome 18. Nine patients continued to term. Neonatal peripheral blood karyotype results were concordant with NIPS results in 7 of these 9 cases (Fig. 5h).

For 8 patients who declined both prenatal and postnatal genetic testing, NIPS results were compared to clinical outcomes to evaluate concordance. Two of the 8 patients (one DS and one 45,X) had fetal ultrasonographic anomalies consistent with the condition suspected by NIPS, declined prenatal diagnostic genetic testing, had an IUFD, and declined postnatal genetic testing (Fig. 5i). These cases were considered concordant, because the probable diagnosis based on fetal anatomy ultrasound abnormalities matched the diagnosis suspected from NIPS results. Four of the 8 patients (3 T18, 1 DS) had normal follow-up fetal anatomy ultrasound results and declined prenatal diagnostic genetic testing (Fig. 5c). Newborn clinical exams performed by pediatricians were normal in all 4 cases, and postnatal genetic testing was not considered indicated. These cases were considered discordant because of the low likelihood that a newborn affected with T18 or DS would be phenotypically normal at delivery. Two patients with DS NIPS results had abnormal follow-up NT results, declined all further services, and opted for TOP (Fig. 5j).

## Discussion

Publications regarding the performance of NIPS have mostly been sponsored by the commercial laboratories performing the test [10, 14, 18, 19, 28]. To our knowledge, this is the largest independent report of clinical experience using NIPS in a tertiary referral center in the United States. During the first two years of offering NIPS, we witnessed the powerful benefits of this technology. First, multiple studies have shown that NIPS has a very high NPV, including a recently published prospective multicenter study that found an NPV of 100 % for DS [19]. Indeed, we are not aware of any normal discordant results among this patient cohort during this timeframe (Fig. 3). Normal NIPS results thus do greatly reduce the probability of a fetus affected with the conditions included in the screen. Whether NIPS is done as a primary screen at 10 weeks gestation or at 22 weeks after abnormal fetal ultrasound findings, normal results provide reassurance. Consequently, our rate of amniocentesis dropped significantly, a trend reported by others [20, 29–31]. In this series, 2 % of patients with normal NIPS results chose prenatal diagnostic genetic testing, as compared to 42 % of patients with abnormal results (Figs. 4 and 5).

Second, NIPS proved to be an invaluable tool for patients wanting to learn as much as possible about fetal health prior to delivery without incurring the miscarriage risk associated with CVS and amniocentesis. These patients commonly expressed that they would not consider TOP, regardless of the diagnosis. Even if NIPS results were abnormal, the family would benefit from being able to prepare for delivery with greater certainty about what lay ahead. In our cohort, this use of NIPS was especially obvious among patients confronted with abnormal findings on their mid-trimester fetal anatomy ultrasound, typically done at 18-22 weeks. Several of our patients in this situation voiced understanding that NIPS results are not diagnostic and still chose NIPS instead of diagnostic genetic testing (Fig. 1). Abnormal fetal ultrasound was the most frequent indication for NIPS (66 %) among patients drawn after 20 weeks.

Our experience also confirmed the limitations of NIPS. First, only a handful of conditions are included in the NIPS panels, so normal results only lower the likelihood of the fetus being affected with those disorders. In this cohort, patient 58 was a 35-year-old who presented at 13 weeks for routine screening. The NT measurement was markedly abnormal. The couple opted for NIPS, which returned with normal results. Fetal ultrasound at 16.3 weeks showed echogenic bowel, a two-vessel cord, unilateral clubfoot, unilateral renal agenesis, and an atrial septal defect. CMA and karyotyping of amniocytes showed mosaicism for trisomy 22, a condition not included in NIPS at that time. Thus, patients with normal NIPS results should still be offered a fetal anatomy ultrasound to screen for congenital abnormalities.

Second, although normal discordant NIPS results are rare with such a high NPV, they do occur. Reports of “false negatives” have started to appear [32, 33], as the number of patients having NIPS has increased. Since data collection ended for this cohort, we have had one normal discordant result in a 34-year-old who presented at 13 weeks for routine screening. The NT measurement was enlarged. NIPS results were normal, but karyotyping of fetal tissue revealed 47,XY,+21 in all cells.

Third, abnormal NIPS results confer a high risk, but not a diagnosis, of a fetal abnormality. The PPV was 77.4 % (95 % CI 63.4 - 87.3) in this cohort for all conditions included in the NIPS panels. We also observed that the PPVs differed in this cohort for each condition included in NIPS. Abnormal results for DS were the most frequent, and these results had the highest PPV. T18 was the next most frequent condition, and results had the second highest PPV. Abnormal results for the combined SCAs and T13 were the least frequent and had the lowest PPVs. This matches trends in the published literature [10, 14, 18, 28, 34]. There may be multiple reasons for this. T13 and T18 are less prevalent than DS, which would adversely impact the PPV. Confined placental mosaicism (CPM) for a trisomic cell line was observed more frequently at CVS for chromosomes 13 and 18 than for chromosome 21 [35, 36]. Postzygotic loss (“trisomy rescue”) in a trophectoderm progenitor cell, leading to placental mosaicism for euploidy, was hypothesized to facilitate the intrauterine survival of T13 and T18 conceptuses [37]. The percentage of placental DNA in maternal circulation is generally lower when the fetus has T18, T13, or 45,X and higher when the fetus has DS [4, 28, 38].

There are several causes of discordant results. Discordant results may be caused by statistical limitations of the analysis algorithms and/or uneven sequencing coverage secondary to guanine and cytosine (GC) content differences between chromosomes [39, 40]. PPV and NPV are influenced by the prevalence of the condition in the screened population. As expected in this cohort, maternal age at delivery was significantly younger among patients with abnormal *discordant* results, compared to patients with abnormal *concordant* results (Fig. 2a). Other studies have also shown a drop in PPV when women of all levels of risk are included, as compared to a solely high-risk population [14, 28]. Normal discordant results have been reported to be more likely at an early gestational age because of a lower placental cfDNA fraction [6, 7]. Gestational age at time of screening was not associated with concordance of results in our series (Fig. 2b), but we do not have data on body mass index and placental cfDNA fraction. Maternal mosaicism was detected in several instances of discordant NIPS results abnormal for an SCA [34, 41] and has also been reported for T18 [21]. Patient 51 (Table 2) was an example of this. Maternal chimera due to prior organ transplant was the likely cause of gender discordance for patient 60 (described above). CPM can cause discordant NIPS results, since the source of non-maternal cfDNA during gestation is the placental cytotrophoblastic cell line. Aneuploidy in the cytotrophoblastic cell line with a diploid fetus may cause an *abnormal* discordant NIPS result, whereas diploidy in the cytotrophoblastic cell line with a trisomic fetus may cause a *normal* discordant NIPS result [32, 42–44]. There are several case reports of discordant NIPS results in pregnancies with proven CPM [33, 45–49]. Placental testing was not done in this cohort, because management is not influenced by results, but CPM may explain the discordant results in patient 46 (Table 2). Other biological reasons for discordant results include a vanishing twin with aneuploidy and maternal metastatic disease [50].

Apparent sex discordance can have a variety of causes, including inaccurate sex assessment on ultrasound, a co-twin demise, the statistical limitations of NIPS, and a fetus affected with one of the various disorders of sexual development. NIPS did not include the option of fetal sex assessment during the entire timeframe reported here, and not all patients chose to learn the predicted fetal sex from NIPS. Nevertheless, we are aware of only one instance of confirmed fetal sex discrepancy, in a patient who had a personal history of a renal transplant from a male donor.

Thus, it is imperative that providers make every effort to confirm NIPS results that are abnormal or appear discordant for fetal sex. Ideally, confirmation would be with diagnostic genetic testing done prior to making any irrevocable decisions about pregnancy management. Twenty-two families (42 %) in this cohort with abnormal NIPS results chose to have prenatal diagnostic genetic testing (Fig. 5a, b, d, e and Fig. 6). Nine patients in this group continued to term and took advantage of all opportunities to gain information about fetal health during their pregnancies. However, patients may decline prenatal diagnostic testing after an abnormal NIPS result, as did 31 (58 %) patients in this series. Patients incorporate other clinical information about fetal status into their decision-making. If serum screen results, NIPS results, and fetal anatomy ultrasound results all suggest the same diagnosis, the patient may not need further confirmation. Or a patient’s concern about the suspected condition may not be sufficient to warrant the risk of the invasive procedures needed for prenatal diagnosis. And many patients pursue prenatal genetic screening to be better prepared for the birth, and would never incur the risk of a prenatal invasive procedure, regardless of NIPS results. Twenty-two patients in this cohort with abnormal NIPS results (41 %) declined prenatal diagnostic genetic testing with the intention of continuing to term (Fig. 5c, f, h, and i).

Counseling patients about abnormal NIPS results is complex. The likelihood of an affected fetus depends on a priori risk and is influenced by what else is known clinically. For example, in this cohort, abnormal NIPS results were more likely to be discordant when fetal anatomy ultrasound was normal (67 %) than when abnormalities were seen (10 %). Determining appropriate follow-up is also complicated. In some cases, doing a CVS to obtain diagnostic genetic test results may be the best approach, but it is problematic for several reasons. Since the non-maternal component of cfDNA during pregnancy is the placental cytotrophoblast, genetic testing via CVS is a repeat analysis of the same tissue type. If CVS reveals mosaicism, amniocentesis is recommended, and the pregnancy is subjected to two invasive procedures. Cytotrophoblasts are used for karyotyping in direct and short-term culture after CVS. Results from long-term CVS culture are generally based on analysis of the villi mesenchymal cores. Recommendations are to analyze both direct and long-term culture for the most accurate results [35, 36]. Even so, CVS may not reveal CPM, as only a small portion of the placenta is biopsied during the procedure. Select portions of the placenta may preferentially release cells into maternal circulation [48], and these may not be the same regions sampled by CVS. Patients should be counseled carefully about these limitations.

Multiple methods for diagnostic genetic testing are available. Karyotyping provides both numerical and structural information and distinguishes free trisomy from an unbalanced Robertsonian translocation. The latter can be inherited, with significantly increased recurrence risks when one parent is a carrier. However, chromosomal microdeletions have been added to NIPS panels, and these are typically undetectable with karyotyping. IFISH and CMA can detect both submicroscopic changes and aneuploidy, and these methods are more successful at obtaining a diagnosis from non-viable, frozen, or formalin-fixed, paraffin-embedded (FFPE) tissue [51]. But IFISH and CMA do not reveal structural rearrangements, such as unbalanced Robertsonian translocations causing DS.

Maternal karyotyping should be offered after abnormal NIPS results to rule out maternal mosaicism, especially when an SCA is suspected. Beyond possibly providing an explanation for abnormal NIPS results in a current pregnancy, patients with mosaicism are not good candidates for using NIPS in subsequent pregnancies.

For the 632 patients undergoing NIPS, 92 % of the tests were performed in one of the four major commercial laboratories offering testing during this timeframe. Although the results mainly reflected one commercial lab doing NIPS, all four of those laboratories were represented in both the normal and the abnormal results groups of the cohort. In addition, the published performance of NIPS is remarkably similar across all test platforms in studies sponsored by the commercial laboratories [6, 9, 10, 12, 14, 19]. Thus, the main emphases of this report, that the PPV of NIPS is less than 100 % and abnormal results should be confirmed by diagnostic testing, apply to all NIPS platforms and methods currently in use.

A limitation of this report is that normal pregnancy outcome was not confirmed for 73 % of patients with normal NIPS results. This precluded us from calculating sensitivity, specificity, or NPV for this cohort. However, the PPV of NIPS, the main focus of this report, is unaffected by outcomes of patients with normal NIPS results. Our center does not routinely collect outcome data for patients referred to us who do not deliver at our hospital, which is not unusual in a busy clinical practice. Based on our past experience with referral providers, we feel it likely that we would have been informed of any normal discordant result uncovered by the birth of a child affected with T13, T18, or DS. However, a normal discordant result could have remained undetected if a patient experienced a spontaneous pregnancy loss, and no postnatal genetic testing was done.

## Conclusions

NIPS for fetal chromosome abnormalities using cfDNA in maternal serum is a powerful tool. It can be of enormous benefit to patients seeking information about fetal health. But with a PPV of less than 100 %, abnormal results cannot be considered diagnostic. Most ordering providers will not have time for a detailed discussion of sensitivity, specificity, PPV, and NPV when talking with patients about prenatal genetic screening options. At a minimum, providers should emphasize that 1) normal discordant results, though rare, do occur and 2) there are multiple possible explanations for an abnormal NIPS result. While an affected fetus is a common cause, it is not the only one. Management of an abnormal NIPS result is complex, and these patients should always be referred to a center offering genetic counseling, high-resolution fetal ultrasound, and diagnostic genetic testing.

### Consent

This study was approved by the Human Subjects Division (Application no. 47,683) and the IRB at the UWMC. The requirement to obtain written consent and the requirement for HIPAA authorization were both waived by the IRB.

## Abbreviations

AFP: alpha-fetoprotein
cfDNA: cell-free deoxyribonucleic acid
CI: confidence interval
CMA: cytogenomic microarray analysis
CPM: confined placental mosaicism
CVS: chorionic villus sampling
DS: Down syndrome
FFPE: formalin-fixed, paraffin-embedded
GC: guanine and cytosine
HIPAA: Health Insurance Portability and Accountability Act
IFISH: interphase fluorescence in situ hybridization
IRB: Institutional Review Board
IUFD: intrauterine fetal demise
NIPS: non-invasive prenatal screening
NPV: negative predictive value
NT: nuchal translucency
PGFT: prenatal genetics and fetal therapy
PPV: positive predictive value
SCA: sex chromosome aneuploidy
SNP: single nucleotide polymorphism
T13: trisomy 13
T18: trisomy 18
TOP: termination of pregnancy
UWMC: University of Washington Medical Center
